# The effect of thriving at work on work-family conflict: the mediating role of workaholism

**DOI:** 10.3389/fpsyg.2023.1136470

**Published:** 2023-11-23

**Authors:** Xudong Ni, Zining Zeng, Jinyu Zhou

**Affiliations:** School of Economics and Management, Zhejiang Sci-Tech University, Hangzhou, China

**Keywords:** thriving at work, workaholism, work-family conflict, work-family separation preference, trust climate

## Abstract

Thriving at work is a relatively new concept in the field of organizational behavior, and many scholars have emphasized the importance of its outcomes in the last decade or so, but we still know little about the possible dark side of thriving at work. In this study, based on the conservation of resources theory, we studied the effect of thriving at work on work-family conflict, the mediating effects of workaholism, and the moderating effects of work-family separation preference and trust climate. By analyzing 372 samples, we found that thriving at work was significantly and positively related to work-family conflict; workaholism partially mediated the relationship between thriving at work and work-family conflict; work-family separation preference negatively moderated the relationship between thriving at work and workaholism. The moderating role of the trust climate was not verified. This paper explores the internal mechanisms by which thriving at work negatively affects the family sphere and helps individuals avoid falling into the dark side of thriving at work.

## 1 Introduction

We live in a fast-paced world characterized by turbulent economic changes in a globalized marketplace where individuals and businesses expect dynamic growth and development to adapt to the changing environment. Thriving at work is a joint experience of a sense of vitality and learning (Spreitzer et al., 2005). Thriving at work is a relatively new concept in the field of organizational behavior, and in the last decade or so, many scholars have emphasized the importance of its outcomes; they found that thriving at work was positively correlated with health (Walumbwa et al., 2017; Jo et al., 2020; Kleine et al., 2022), commitment (Porath et al., 2012; Abid et al., 2019; Zhai et al., 2022), job satisfaction (Chang and Busser, 2020; Jiang et al., 2020; Okros and Vîrgă, 2022), work well-being (Qaiser et al., 2018; Basinska and Rozkwitalska, 2020), creative performance (Wallace et al., 2016; Christensen-Salem et al., 2020) and negatively correlated with turnover intentions (Ren et al., 2015; Kleine et al., 2019; Chang and Busser, 2020). In addition, some scholars have examined the positive associations between thriving at work and the family domain (Russo et al., 2015; Carmeli and Russo, 2016; Xu et al., 2020). The antecedents and positive results of thriving at work have been discussed by many scholars. However, we still know very little about the possible dark side of thriving at work. Halbesleben et al. (2009) suggested that if a person was thriving at work, he might neglect some non-work aspects. Porath et al. (2012) argued that those who are thriving at work might invest more energy in their work, but this was detrimental to their thriving outside of work; their findings also showed that despite some spillover effects between thriving at work and non-work, the two types of thriving were different. Chinese scholars Han and Wei (2013) suggested exploring the possible adverse effects of thriving at work in certain contexts. Although these ideas have been previously proposed, few scholars have investigated the possible dark side of thriving at work.

work-family conflict reflects the incompatibility of some aspects of the simultaneous pressures from work and family (Greenhaus and Beutell, 1985), with the essence being the misalignment of resources.^1^ According to the conservation of resources theory (Hobfoll, 1989), a resource applied to one domain leads to a reduction in another domain; therefore, when employees are motivated to invest too much time and energy in their work by a sense of vitality and learning implied by thriving at work, they become workaholics, causing a relative decrease in the investment of resources in the family domain. Workaholics tend to work longer and harder than others, may often miss family activities in the evening or on weekends, and always bring work home. They also tend to blur the boundaries between work and non-work through recreational activities that promote or complement work, and they even continue to overwork when facing negative marital or health outcomes (Ng et al., 2007), causing work-family conflict.

Whether thriving at work leads to workaholism is clearly influenced by various factors. Based on boundary theory (Nippert-Eng, 1998), Kim (2019) argued that intensive work behavior promoted workaholism through habit formation, and individual preferences for work-family boundary delineation influenced the actual work time of individuals and could disengage employees from repeated work; therefore, it can be speculated that disengaging employees who are thriving at work away from work during non-work time may be effective in avoiding workaholism. In addition, as an important environmental resource, trust climate may positively impact the work attitudes of employees and can reduce the perception of threat and hostility in the work environment for employees (Mayer et al., 1995). Therefore, when dealing with difficulties and challenges in the work process, trust climate can make individuals more willing to seek help from colleagues and make employees feel more comfortable delegating tasks to colleagues, thus reducing their working hours and work stress and avoiding workaholism. Hence, at the individual and environmental levels, work-family separation preference and trust climate are regarded as moderating variables in the relationship between thriving at work and workaholism.

As a positive experience that encompasses a sense of vitality and learning, thriving at work is beneficial to the growth of individuals in the workplace. To achieve long-term individual thriving at work and weaken the pathways of its negative effects, its dark side should be studied. Based on this, in this study, we investigated the effects of thriving at work on work-family conflict, the mediating effects of workaholism and the moderating effects of work-family separation preference and trust climate to explore the mechanisms underlying the negative effects of thriving at work on the family domain and help individuals avoid falling into the dark side of thriving at work.

### 1.1 Thriving at work and work-family conflict

Thriving at work refers to a joint experience of vitality and learning in the work domain (Spreitzer et al., 2005). A high sense of vitality and learning associated with thriving at work promotes job performance (Frazier and Tupper, 2016; Walumbwa et al., 2017). From a psychological perspective, it increases job satisfaction (Chang and Busser, 2020). Therefore, when people perceive work activities to be personally beneficial, they are more likely to devote more resources to work and less to family. According to the work-family resource model proposed by Ten Brummelhuis and Bakker (2012), too many personal resources invested in one role can negatively affect the extent to which the needs and goals of the role in other domains are met, i.e., lead to work-nonwork conflict. Hence, our first hypothesis is as follows.

*Hypothesis 1:* Thriving at work can lead to work-family conflict.

### 1.2 Mediating effects of workaholism

Since the concept of workaholism was introduced as an academic topic, many scholars have attempted to define it more clearly. Although these definitions are still inconsistent, they basically reflect two core elements, i.e., work overload and internal work drive (Balducci et al., 2012). Therefore, in this paper, the definition of Clark et al. (2014) is used. They defined workaholism as the tendency to work compulsively and excessively, i.e., not only working for long hours but also persistently and frequently thinking about work during non-working hours and working beyond reasonable expectations when individuals feel compelled to work due to internal pressures. That is, workaholism reflects the addiction of employees to work, a compulsive or persistent and irresistible state of work.

From a cognitive perspective, thriving at work facilitates job performance (Frazier and Tupper, 2016; Walumbwa et al., 2017), contributing to self-efficacy. Some scholars have argued that individuals are prone to being workaholics when their self-efficacy is too high because they believe they are ideally suited to handle work (Ng et al., 2007). From an affective perspective, thriving at work is positively related to positive affect (Porath et al., 2012), job satisfaction (Chang and Busser, 2020), etc. According to the conservation of resources theory (Hobfoll, 1989), the increase in positive affective resources makes employees tend to continue to devote their time and other resources to work. From a behavioral perspective, the need for individuals to think and improve repeatedly during learning motivates individuals to actively invest more resources, such as time, in their work. Evidence showed that vitality was an important dimension of thriving at work and could stimulate work initiative (Carmeli et al., 2009). Han and Wei (2013) suggested that employees thriving at work exhibited more extra-role behavior and were, therefore, likely to overstep their authority and complete tasks not belonging to them tasks, which directly results in longer working time. Therefore, we can assume that people who are thriving at work may tend to be intrinsically compulsive and overworked, which is manifested by an increase in workaholism. Thus, we present the following hypothesis.

*Hypothesis 2:* Thriving at work is positively related to workaholism.

Workaholism manifests compulsive and excessive work, usually characterized by a tilt of time, psychological, emotional and other resources toward work. Employees with workaholic tendencies have a strong emotional drive to continue working, making it difficult for them to leave the workplace and even to integrate into the family. Bakker et al. (2013) also argued that workaholics could hardly relax even in their leisure time and were unable to effectively balance their work and family roles. Thus, according to the conservation of resources theory (Hobfoll, 1989), excessive commitment to work leaves the resource demands of family roles unmet, which in turn causes work-family conflict. Hence, we propose the following hypotheses.

*Hypothesis 3:* Workaholism is positively related to work-family conflict.

*Hypothesis 4:* Thriving at work increases work-family conflict through workaholism.

### 1.3 Moderating effects of work-family separation preference and trust climate

Boundary management theorists argued that individuals differed in their preferences for managing work and family boundaries, with some preferring to integrate work and family boundaries (i.e., low level of work-family separation preference) and constructing more permeable boundaries, and others preferring to separate work and family boundaries (i.e., high level of work-family separation preference; Ashforth et al., 2000). Individuals with a high level of work-family separation preference rarely think about and engage in work-related activities while at home. Kim (2019) argued that intensive work behavior promoted workaholism through habit formation, whereas individuals with high levels of work-family separation preference removed themselves from work after hours and inhibited work inertia, effectively reducing the likelihood of workaholism. In addition, individuals with high levels of work-family separation preference can distinguish their work roles from their family roles and adopt relatively different behavioral patterns in the two domains, effectively avoiding reinforcing their work roles through repetitive thinking and actions. However, work-exuberant individuals with low levels of work-family separation preference are driven by the sense of vitality and learning brought by work, with work time extended at home. Even more importantly, from the perspective of stress theory, the burden of multiple roles for one individual tends to cause stress, creating a stressful psychological state and reducing efficiency in dealing with family matters. According to the conservation of resources theory, a low level of work-family separation preference means that employees spend time and energy on work at home as well, and the work-family resource allocation mechanism is further disrupted. As a result, individuals tend to invest fewer low-yield resources in the family domain and more resources in work to obtain compensation. However, at this time, work motivation is no longer dominated by the positive influence of thriving at work but by intrinsic pressure, thus causing workaholism. Hence, we present the following hypothesis.

*Hypothesis 5:* Work-family separation preference moderates the positive relationship between the thriving at work of employees and workaholism. A higher level of work-family separation preference indicates a weaker relationship between thriving at work and workaholism.

According to positive psychology, trust is based on positive expectations of the behavior of the other person and a highly directed relationship or psychological state that the trusting person maintains with the other person. When individuals feel trust and respect, they more easily believe that they are valuable members of the organization, contributing to their self-esteem and self-efficacy so that they do not need to maintain their self-worth through excessive work behavior (Graves et al., 2012). This result is consistent with that of known research, i.e., individuals with lower self-esteem are more likely to be work-obsessed because the outcomes of hard work are more certain and evident than uncertain aspects of life (Ng et al., 2007). The trust climate in the organization makes employees feel comfortable delegating tasks to colleagues reasonably, reducing time spent on work and leaving extra resources for other areas of life. In addition, from the perspective of stress coping, this effective delegation of work tasks is one of the important behavioral ways to relieve work stress. Hence, we propose the following hypothesis.

*Hypothesis 6:* The trust climate in the organization moderates the positive relationship between the thriving at work of employees and workaholism. A stronger trust climate in the organization indicates a weaker relationship between thriving at work and workaholism.

**Figure 1:**
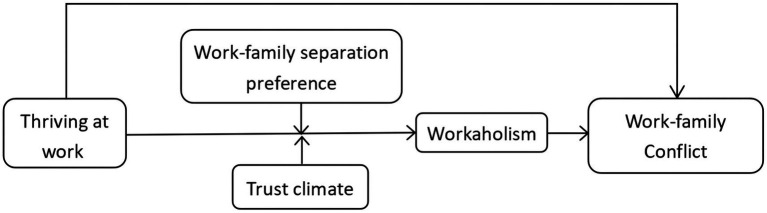
Theoretical model of thriving at work affecting work-family conflict.

The theoretical model in this study is shown in Figure 1.

## 2 Methods

### 2.1 Samples and procedure

In this study, electronic questionnaires were randomly distributed online through social software and professional survey platforms. The respondents were married employed people from 31 provinces in China, including Zhejiang, Shanghai, Hunan, and Jiangsu. We asked employees to report their thriving at work, work-family conflict, workaholism, work-family separation preference and trust climate. A total of 570 anonymous electronic questionnaires were collected in this survey. After eliminating the invalid questionnaires with missing answers and too many similar options, 372 valid questionnaires were obtained, with an effective recovery rate of 65.26%. The composition of the valid samples is shown in Table 1. It can be seen that the samples have a wide distribution and meet the basic requirements of the study. The basic information of the questionnaire respondents is as follows: In terms of gender, 48.1% of the participants were female; 56.7% were aged 31–40; 70.4% with only one child; 73.4% held a bachelor’s degree; 49.7% with 5–10 years of service; and 69.4% of participants were basic staff, grassroots manager/junior title.

**Table 1:** Composition of valid samples.

Name	Category	Number	percent (%)	Cumulative percent (%)
Gender	Male	193	51.88	51.88
	Female	179	48.11	100
Age	30 years old and below	123	33.06	33.06
	31–40 years old	211	56.72	89.78
	41–50 years old	23	6.18	95.96
	51 years old and above	15	4.03	100
Number of children	0	37	9.99	9.99
	1	262	70.43	80.42
	2	69	18.54	98.96
	More than 2	4	1.08	100
Education	High school or below	35	9.41	9.41
	Junior college	40	10.75	20.16
	Bachelor	273	73.38	93.54
	Master or above	24	6.45	100
Service year	2 years and below	23	6.18	6.18
	2–5 years	114	30.65	36.83
	5–10 years	185	49.73	86.56
	10 years and above	50	13.44	100
Rank	Basic staff, grassroots manager/junior title	258	69.35	69.35
	Middle-level manager/intermediate title	99	26.61	95.96
	Senior management/senior title	15	1.08	100

### 2.2 Measures

The scales used in this study were all well-established scales widely used in China. We used a seven-point Likert-type scale ranging from one (strongly disagree) to seven (strongly agree).

#### 2.2.1 Thriving at work

We used the scale developed by Porath et al. (2012) to measure thriving at work, with 10 items. There were five items for learning, such as “I find myself learning often,” and five items for vitality, such as “I feel alive and vital.” Questions 4 and 8 were reverse-scoring questions, which were reverse-scored in the data analysis.

#### 2.2.2 Work-family conflict

The WFC subscale developed by Netemeyer et al. (1996) was used to measure work-family conflict with five items, such as “The demands of my work interfere with my home and family life.”

#### 2.2.3 Workaholism

We used the Dutch workaholism scale (DUWAS) developed by Schaufeli et al. (2009) to measure workaholism. There were 10 items, including 5 items on working excessively, such as “I seem to be in a hurry and racing against the clock,” and 5 items on working compulsively, such as “Working hard is important to me even when I do not enjoy what I am doing.”

#### 2.2.4 Work-family separation preference

The scale developed by Kreiner (2012) was used to measure work-family separation preference, with four items, such as “I do not like to have to think about work while I am at home.”

#### 2.2.5 Trust climate

We measured the trust climate using the scale developed by De Jong and Elfring (2010) with five items, such as “I can count on my team members for help if I have difficulties at work.”

#### 2.2.6 Control variables

The control variables included gender, age, number of children, education level, service year, occupation, and rank.

## 3 Results

### 3.1 Reliability and validity analysis

In this study, reliability analysis was conducted using Cronbach α values. As shown in Table 2, the α coefficients for the learning and vitality subscales of thriving at work were 0.767 and 0.809, and the α coefficients for the working excessively and working compulsively subscales of workaholism were 0.790 and 0.709. The overall α coefficients of the five variables are 0.868, 0.837, 0.869, 0.845, and 0.842, and the reliability coefficients of all scales achieve good reliability of 0.7 or higher, meeting the needs of the study.

**Table 2:** Cronbach α coefficient for each scale.

Variables	Number of items	Cronbach α
Thriving at work	10	0.868
Learning	5	0.767
Vitality	5	0.809
Workaholism	10	0.837
Working excessively	5	0.790
Working compulsively	5	0.709
work-family conflict	5	0.869
Work-family separation preference	4	0.845
Trust climate	5	0.842

We used AMOS 24.0 to conduct confirmatory factor analysis on the five variables (thriving at work, workaholism, work-family conflict, work-family separation preference and trust climate). The results are shown in Table 3. Compared with other alternative models, the five-factor model fits the actual data most satisfactorily (*ꭓ*^2^/df = 1.759, RMSEA = 0.045, IFI = 0.921, TLI = 0.914, and CFI = 0.921), indicating the good discriminant validity of the variables in this study.

**Table 3:** Confirmatory factor analysis.

Model	*ꭓ*^2^/df	RMSEA	IFI	TLI	CFI
Five-factor model (F1, F2, F3, F4, F5)	1.759	0.045	0.921	0.914	0.921
Four-factor model (F1, F2 + F3, F4, F5)	2.170	0.056	0.878	0.867	0.877
There-factor model (F1 + F5, F2 + F3, F4)	2.997	0.073	0.790	0.773	0.788
Two-factor model (F1 + F5, F2 + F3 + F4)	4.122	0.092	0.670	0.646	0.668
Single-factor model (F1 + F2 + F3 + F4 + F5)	5.981	0.116	0.472	0.435	0.469

F1 = thriving at work; F2 = workaholism; F3 = work-family conflict; F4 = work-family separation preference; F5 = trust climate.

### 3.2 Common variance tests

In this study, we further used AMOS 24.0 to test a two-factor model based on an ex ante control for the questionnaire design (anonymity of the questionnaire and reverse scoring of some items). A “five-factor” model was built based on five variables, and the fit index was derived. Next, the method factor, which can explain the common variation of the question items, was added to the model, constituting a “five-factor + method factor” model. If the fit index does not change dramatically from the original model, it indicates no significant common method bias in the measurement. As shown in Table 4, △RMSEA = 0.005, △CFI = −0. 02, and △IFI = -0. 021, indicating that the model was not significantly improved by adding the method factor, and there was no significant common method bias in the measurement.

**Table 4:** Model test fit index.

Fit Index	SRMR	RMSEA	CFI	IFI
“Five-factor” model	0.057	0.045	0.921	0.921
“Five-factor + method factor” model	0.044	0.040	0.941	0.942
Variation	0.013	0.005	−0.02	−0.021

### 3.3 Descriptive statistics and correlation analysis

As shown in Table 5, the correlation analysis reveals significant and positive correlations of thriving at work with workaholism (*r* = 0.40, *p* < 0.01) and work-family conflict (*r* = 0.36, *p* < 0.01) and significant and positive correlations of workaholism with work-family conflict (*r* = 0.63, *p* < 0.01). These results showed that the correlation analysis initially supported the research hypotheses of this paper.

**Table 5:** Descriptive statistics and correlation analysis of variables.

	*M*	*SD*	Thriving at work	Workaholism	Work-family conflict	Work-family separation preference	Trust climate
Thriving at work	5.42	0.79	1				
Workaholism	4.60	0.79	0.40^**^	1			
Work-family conflict	4.82	1.16	0.36^**^	0.63^**^	1		
Work-family separation preference	5.73	1.05	0.06	−0.08	−0.01	1	
Trust climate	4.57	0.96	0.45^**^	0.08	0.13^*^	0.15^**^	1

* *p* < 0.05;

** *p* < 0.01.

### 3.4 Hypothesis tests

#### 3.4.1 Main and mediating effect tests

We tested the mediating effect of workaholism on the relationship between thriving at work and work-family conflict using Model 4 in PROCESS (Model 4 is a simple mediation model), controlling for gender, age, number of children, education level, service year, occupation, and rank. The results (Table 6) showed that the effect of thriving at work on work-family conflict was significant (*β* = 0.51, *t* = 6.85, *p* < 0.01), and the direct predictive effect of thriving at work on work-family conflict remained significant when workaholism was put in (*β* = 0.18, *t* = 2.62, *p* < 0.01). The positive predictive effect of thriving at work on workaholism was significant (*β* = 0.40, *t* = 7.95, *p* < 0.01), and the positive predictive effect of workaholism on work-family conflict was also significant (*β* = 0.85, *t* = 13.11, *p* < 0.01). Furthermore, Table 7 shows that the upper and lower limits of the bootstrap 95% confidence interval for the direct effect of thriving at work on work-family conflict and the mediating effect of workaholism do not contain 0. These results indicated that thriving at work not only directly predicted work-family conflict but also predicted work-family conflict through the mediating effect of workaholism. This direct effect (0.176) and mediating effect (0.338) accounted for 34.19 and 65.79% of the total effect (0.514), respectively. Hypotheses 1, 2, 3, and 4 were validated.

**Table 6:** A mediator model test of workaholism.

Predictive variables	Result Variables					
	Work-family conflict		Workaholism		Work-family conflict	
	*B*	*t*	*B*	*t*	*B*	*t*
Gender	−0.27	−2.32	−0.11	−1.46	−0.17	−1.81^*^
Age	−0.004	−0.04	−0.012	−0.18	0.01	0.08
Number of children	0.11	0.99	0.18	2.52^*^	−0.05	−0.53
Education level	−0.05	−0.57	−0.05	−0.76	−0.01	−0.18
Service year	−0.13	−1.59	−0.07	−1.19	−0.08	−1.11
Occupation	−0.08	−1.30	−0.01	−0.20	−0.08	−1.44
Rank	0.03	0.31	−0.03	−0.35	0.06	0.61
Thriving at work	0.51	6.85^**^	0.40	7.95^**^	0.18	2.62^**^
Workaholism					0.85	13.11^**^
R	0.40		0.43		0.67	
R^2^	0.16		0.19		0.43	
F	8.40^**^		10.35^**^		30.07^**^	

* *p* < 0.05;

** *p* < 0.01.

**Table 7:** Breakdown of total, direct, and mediating effects of workaholism.

	Effect	Boot standard errors	Boot CI lower limit	Boot CI upper limit	Effectiveness ratio
Total effect	0.513	0.04	0.15	0.30	
Direct effect	0.176	0.09	0.004	0.34	34.19%
Mediating effect of workaholism	0.338	0.05	0.24	0.44	65.79%

Boot standard error, Boot CI lower limit and Boot CI upper limit refer to the standard error and the lower and upper limits of 95% confidence intervals of indirect effects estimated by the bias-corrected percentile Bootstrap method, respectively.

#### 3.4.2 Moderating effect test

The moderating effects of work-family separation preference and trust climate on the relationship between thriving at work and workaholism were tested using Model 7 in PROCESS (In Model 7, it is assumed that the first half of the mediation model is moderated), controlling for gender, age, number of children, education level, service year, occupation, and rank. The results of Models 1 and 2 are shown in Table 8. After putting the work-family separation preference and trust climate into the models, the product term of thriving at work and work-family separation preference had a significant predictive effect on workaholism (*β* = −0.11, *t* = −3.06, *p* < 0.01), indicating that work-family separation preference could moderate the predictive effect of thriving at work on workaholism; the product term of thriving at work and trust climate had a non-significant predictive effect on workaholism (*β* = 0.01, *t* = 0.33, *p* = 0.74), suggesting that trust climate did not moderate the relationship between thriving at work and workaholism.

**Table 8:** Gender, work-family separation preference, and trust climate moderating effects test (*n* = 372).

Predictive variables	Workaholism			
	Model1		Model2	
	*B*	*t*	*B*	*t*
Gender	−0.11	−1.45	−0.12	−1.61
Age	−0.01	−0.17	−0.03	−0.45
Number of children	0.17	2.33^*^	0.17	2.41^*^
Education level	−0.05	−0.83	−0.06	−0.95
Service year	−0.06	−1.16	−0.07	−1.24
Occupation	0.002	0.05	−0.02	−0.54
Rank	−0.03	−0.41	−0.01	−0.10
Thriving at work	1.04	4.87^**^	0.40	2.07^**^
Work-family separation preference	0.53	2.68^*^		
Thriving at work × Work-family separation preference	−0.11	−3.06^*^		
Trust climate			−0.20	−0.82
Thriving at work × Trust climate			0.01	0.33
R	0.46		0.45	
R^2^	0.21		0.20	
F	9.78^**^		9.06^**^	

* *p* < 0.05;

** *p* < 0.01.

**Figure 2:**
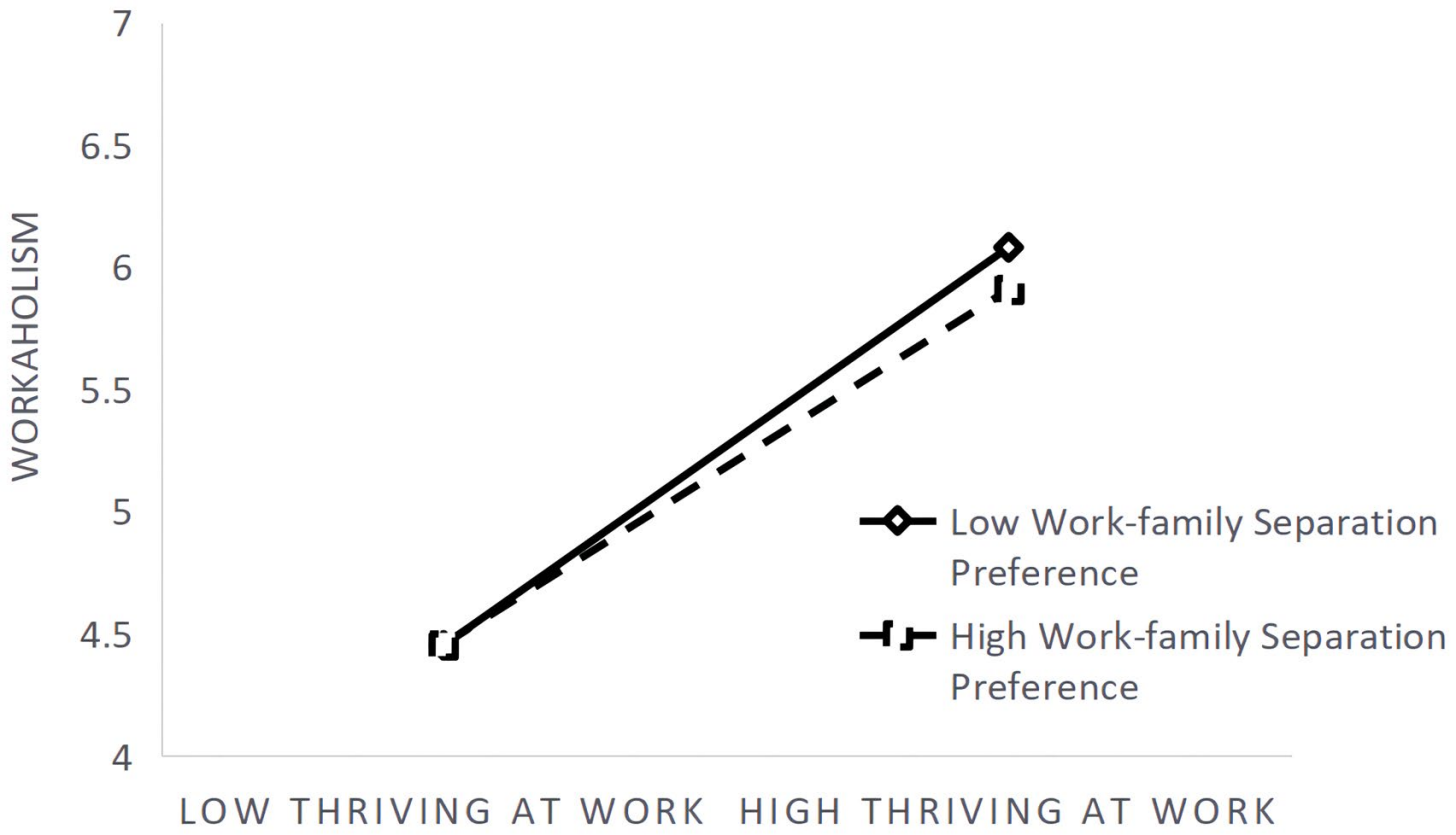
Moderating effect of work-family separation preference in the relationship between thriving at work and workaholism.

Further simple slope analysis of the moderating effect of work-family separation preference (Figure 2) showed that thriving at work was a significant predictor of workaholism for subjects with low levels of work-family separation preference (M-1SD; simple slope = 0.51, *t* = 8.44, *p* < 0.01), whereas for subjects with high levels of work-family separation preference (M + 1SD),its predictive effect was smaller (simple slope = 0.27, *t* = 4.15, *p* < 0.01), indicating that as the level of individual work-family separation preference increased, the predictive effect of thriving at work on workaholism tended to decrease. Hypothesis 5 was verified, but Hypothesis 6 was not.

## 4 Discussion

In this study, the impact of thriving at work on work-family conflict was investigated based on the conservation of resources theory. The study empirically supported the adverse effect of thriving at work in triggering work-family conflict and revealed the partially mediating role of workaholism in the relationship between thriving at work and work-family conflict. We also found that work-family separation preference negatively moderated the relationship between thriving at work and workaholism. The moderating effect of trust climate was not verified.

### 4.1 Theoretical implications

First, the specific mechanisms of the dark side of thriving at work were analyzed in depth. Current empirical studies on thriving at work are more often from the perspective of its positive effects, and few scholars have explored its possible dark side, although some have questioned and appealed to this issue (Han and Wei, 2013). It has been shown that although work is often perceived as a healthy and beneficial activity when commitment to it reaches an unhealthy degree, its positive meaning can be reduced (Bakker et al., 2011). In this study, we proposed a theoretical pathway by which thriving at work affects work-family conflict through workaholism and its moderating variables. This pathway suggests that the positive work states of certain individuals may be transformed into negative family outcomes under certain circumstances, and the proposed model contributes to the theoretical study of the dark side of thriving at work.

Second, this paper can provide insight into how to achieve long-term thriving at work. Spreitzer et al. (2005) suggested that future research should focus on how individuals maintain their thriving over time. The negative family aspects of workaholism may be one reason why thriving is not sustained. Previous research showed that human vitality was depleted when individuals got into potentially challenging, frustrating, exasperating, or rude relationships (Fritz et al., 2011). Thus, employees caught in negative family relationships may have diminished vitality and thus lose their sense of thriving at work (Spreitzer et al., 2005). This paper verified the negative moderating effect of work-family separation preference on the relationship between thriving at work and workaholism, showing that individuals with high levels of work-family separation preference could avoid workaholism while maintaining a high degree of thriving, which can help them to maintain good work states and family functioning. The moderating effect of trust climate was not verified. However, we believe that, as suggested in our hypothesis, trust climate makes individuals feel comfortable delegating tasks to colleagues and spend less time on work; in addition, the combined effect of trust climate and thriving at work enables individuals to advance their work more smoothly, making them satisfied with their work environment and thus more prone to becoming “workaholics.” The difference in the size of the two effects leads to uncertainty in the study results.

Third, this paper fills a gap in the field of work-family conflict by clarifying that even positive work states can be an antecedent of work-family conflict from a resource-based perspective. In recent years, the literature on the work-family domain has become increasingly rich, with the exploration of the antecedents of work-family conflict focusing on three categories, including work domain (job flexibility, etc.), non-work domain (social support, etc.), and demographic and individual variables (gender, personality traits, etc.; Carlson and Perrewé, 1999; Byron, 2005; Michel et al., 2011; French et al., 2018). In the work domain, through numerous empirical studies, scholars have conducted meta-analyses of the relationship between variables, such as job demands, job control, job role overload, job role flexibility and work-family conflict (Liao et al., 2019). However, we found few scholars exploring the effect of positive work psychological states on work-family conflict. In this study, we studied the mechanism underlying thriving at work, by which a positive work state can be transformed into workaholism and affect individual resource allocation, thereby causing work-family conflict.

### 4.2 Practical implications

For individuals, thriving at work is a positive state that includes a sense of vitality and learning and contributes to their professional development. However, as the saying goes, “too much is as bad as too little.” Work needs to be moderately done. To make thriving at work better for personal growth, individuals can separate work and family to avoid workaholism-caused work-family by the following methods. First, manage time well and complete work-family time allocation. Time management tools, such as Gantt charts, can be used to improve productivity (Goktug et al., 2013). All work can be completed during working hours as much as possible. Work notifications on electronic devices can be turned off when at home. Second, actively communicate with family to improve the emotional regulation ability and avoid bringing work stress into the family. Third, clarify and distinguish work and family roles. Unlike being serious and rigorous at work, at home as parents and partners, individuals need to be careful and gentle in maintaining family relationships and strengthening emotional care and communication with family members (Kim et al., 2019). Individuals should try not to think about work when spending time with family members to avoid inertia in thinking and behavior.

For companies, workaholism is not always a good thing. Porter (2001) found that workaholics could negatively affect the organization because they invested too much time and energy in their work, created excessive standards for team members, and did everything themselves due to distrust of and competition with their peers, sometimes even hindering the completion of team tasks. Therefore, companies should take some measures, such as regularly observing their behavior and organizing training to guide them in maintaining a work-family balance, to stop employees from becoming workaholics. Companies should also respect the work-family separation preference of employees and adopt different management strategies for employees with different types of preference. For individuals with low levels of work-family separation preference, family-friendly policies, such as allowing them to telecommute and providing more flexible working hours, can be implemented. For individuals with high levels of work-family separation preference, companies should respect their right of “disconnection,” which has been proven to pay dividends to organizations by improving work performance (Kalliath et al., 2020). Therefore, work-life separation policies and practices can be offered at the organizational level.

### 4.3 Limitations and future research

This study has the following limitations. First, a self-report approach was used for the questionnaire in this study, which may lead to common variance. Second, the study did not further differentiate between the different dimensions of variables such as thriving at work and workaholism, which directly affects the internal validity of the research. Third, the moderating effect of trust climate was not validated in this study, but we still believe that trust climate could have an impact on the relationship between individuals’ thriving at work and workaholism. An interview study with nurses found that a work climate of trust and respect was an important external factor in promoting employees’ thriving at work (Jackson, 2022). Therefore, trust climate may make individuals more satisfied with the work environment, leading to a tendency to spend time on work and becoming workaholics. On the other hand, we speculate that the overall work saturation of the team should also be taken into account, as human time and energy are limited, and employees will choose to actively help their colleagues on the basis of ensuring that their work can be effectively completed (Deshon et al., 2004). When the overall workload of the team is very high, even though colleagues trust each other, they cannot help others to solve problems if they cannot take care of themselves. This will be further analyzed in the future.

Many scholars have incorporated affective experience dimensions into the definition of workaholism, for example, Spence and Robbins (1992) viewed workaholism as a three-dimensional conceptualization encompassing work involvement, work drive, and work enjoyment. Differences in affective experience may have various effects on both work experience and work-family conflict. For example, employees with high levels of work enjoyment may be addicted to work because of this positive experience and may alleviate work-family conflict because of the spillover of the positive emotions into the family domain. This study adopted the two-dimensional definition of Clark et al. (2014) and did not consider its affective dimension, so further segmentation of workaholism and empirical research could be conducted accordingly in the future. In addition, the moderating variables need to be further added based on the present model in the future. The possible effects of factors, such as job type and family climate, can be studied, and variables in other domains outside of work and family domains may also be a possible exploration direction. Finally, based on the conservation of resources theory, we believe that the positive effects originating from work may permeate other areas of life (Aziz et al., 2010). Therefore, the combined effects of thriving at work on other areas of life must be investigated, and how to achieve a win-win situation in both work and non-work areas can be explored.

## Data availability statement

The original contributions presented in the study are included in the article/Supplementary material, further inquiries can be directed to the corresponding author.

## Ethics statement

The studies involving humans were approved by Zhejiang Sci-Tech University, China. The studies were conducted in accordance with the local legislation and institutional requirements. The participants provided their written informed consent to participate in this study.

## Author contributions

XN: conceptualization, supervision, and project administration. ZZ: methodology, data analysis, and writing. JZ: review and editing. All authors contributed to the article and approved the submitted version.

## Footnotes

^1^Greenhaus and Beutell further pointed out that work–family conflict had a two-way character. The interference with family-related responsibilities due to the time and pressure of work is a work-to-family conflict, while the interference with work-related responsibilities due to family-related demands is a family-to-work conflict. Considering the scope of the study, the work–family conflict in this paper only refers to the former, i.e., work-to-family conflict.
