# Supplementary material for: The effect of thriving at work on work-family conflict: the mediating role of workaholism
Source: Front Psychol. 2023 Nov 23;14:1136470. doi: 10.3389/fpsyg.2023.1136470 (PMC10702575; doi:10.3389/fpsyg.2023.1136470)
Supplement: Supplementary file 1 [file Table_1.DOCX]

**Appendix: Questionnaire**

Dear Ms./Mr.

Hello. Thank you for taking the time to complete this questionnaire. This questionnaire aims to explore the mechanism by which the thriving at work of employees affects work-family conflict and is for academic research purposes only. In addition, there are no good or bad options, and the researcher will keep the information in each questionnaire strictly confidential, so please answer it according to your situation. It will take approximately 10 minutes to complete the questionnaire. Thank you for filling it out!

**Part I: Basic personal information**

1. Your gender.

①Male ② Female

1. Your age.

①30 years old and below ②31–40 years old

③41–50 years old ④51 years old and above

1. Your marital status.

①Married ②Unmarried ③Other

1. The number of your children.

①0 ②1 ③2 ④More than 2

1. Your education level.

①High school and below ②Junior college ③Bachelor

④Master or above

1. Your service year (time in the company).

①2 years and below ②2–5 years ③5–10 years

④10 years and above

1. Your current occupation.

①Corporate employee ②Medical practitioner ③Lawyer

④Freelancer ⑤Entrepreneur ⑥Other

1. Your current rank.

①Basic staff, grassroots manager/junior title

②Middle-level manager/intermediate title

③Senior management/senior title

**Part II Thriving at Work Assessment**

| Questions (evaluation criteria are as follows)  (1--Strongly disagree 2--Disagree 3--Rather disagree 4--Fair  5 - Rather agree 6--Agree 7--Strongly Agree) | 1 | 2 | 3 | 4 | 5 | 6 | 7 |
| --- | --- | --- | --- | --- | --- | --- | --- |
| 1、I find myself learning often |  |  |  |  |  |  |  |
| 2、I continue to learn more and more as time goes by |  |  |  |  |  |  |  |
| 3、I see myself continually improving |  |  |  |  |  |  |  |
| 4、I am not learning (R) |  |  |  |  |  |  |  |
| 5、I have developed a lot as a person |  |  |  |  |  |  |  |
| 6、I feel alive and vital |  |  |  |  |  |  |  |
| 7、I have energy and spirit |  |  |  |  |  |  |  |
| 8、I do not feel very energetic (R) |  |  |  |  |  |  |  |
| 9、I feel alert and awake |  |  |  |  |  |  |  |
| 10、I am looking forward to each new day |  |  |  |  |  |  |  |

**Part III Workaholism Assessment**

| Questions (evaluation criteria are as follows)  (1--Strongly disagree 2--Disagree 3--Rather disagree 4--Fair  5 - Rather agree 6--Agree 7--Strongly Agree) | 1 | 2 | 3 | 4 | 5 | 6 | 7 |
| --- | --- | --- | --- | --- | --- | --- | --- |
| 1、I seem to be in a hurry and racing against the clock. |  |  |  |  |  |  |  |
| 2、I find myself continuing to work after my coworkers have called it quits. |  |  |  |  |  |  |  |
| 3、I stay busy and keep many irons in the fire. |  |  |  |  |  |  |  |
| 1. I spend more time working than on socializing with friends, on hobbies, or on leisure activities. |  |  |  |  |  |  |  |
| 5、I find myself doing two or three things at one time such as eating lunch and writing a memo, while taking on the telephone. |  |  |  |  |  |  |  |
| 6、 It is important to me to work hard even when I do not enjoy what I am doing. |  |  |  |  |  |  |  |
| 7、I feel that there is something inside me that drives me to work hard. |  |  |  |  |  |  |  |
| 8、I feel obliged to work hard, even when it is not enjoyable. |  |  |  |  |  |  |  |
| 9、I feel guilty when I take time off work. |  |  |  |  |  |  |  |
| 10、It is hard for me to relax when I am not working. |  |  |  |  |  |  |  |

**Part IV Work-Family Conflict Assessment**

| Questions (evaluation criteria are as follows)  (1--Strongly disagree 2--Disagree 3--Rather disagree 4--Fair  5 - Rather agree 6--Agree 7--Strongly Agree) | 1 | 2 | 3 | 4 | 5 | 6 | 7 |
| --- | --- | --- | --- | --- | --- | --- | --- |
| 1、The demands of my work interfere with my home and family life. |  |  |  |  |  |  |  |
| 2、The amount of time my job takes up makes it difficult to fulfill family responsibilities. |  |  |  |  |  |  |  |
| 3、Things I want to do at home do not get done because of the demands my job puts on me. |  |  |  |  |  |  |  |
| 4、My job produces strain that makes it difficult to fulfill family duties. |  |  |  |  |  |  |  |
| 5、Due to work-related duties, I have to make changes to my plans for family activities. |  |  |  |  |  |  |  |

**Part V. Work-family Separation Preference Assessment**

| Questions (evaluation criteria are as follows)  (1--Strongly disagree 2--Disagree 3--Rather disagree 4--Fair  5 - Rather agree 6--Agree 7--Strongly Agree) | 1 | 2 | 3 | 4 | 5 | 6 | 7 |
| --- | --- | --- | --- | --- | --- | --- | --- |
| 1、I don’t like to have to think about work while I’m at home |  |  |  |  |  |  |  |
| 2、I don’t like work issues creeping into my home life |  |  |  |  |  |  |  |
| 3、I prefer to keep work life at work |  |  |  |  |  |  |  |
| 4、I like to be able to leave work behind when I go home |  |  |  |  |  |  |  |

**Part VI Organizational Trust Climate Assessment**

| Questions (evaluation criteria are as follows)  (1--Strongly disagree 2--Disagree 3--Rather disagree 4--Fair  5 - Rather agree 6--Agree 7--Strongly Agree) | 1 | 2 | 3 | 4 | 5 | 6 | 7 |
| --- | --- | --- | --- | --- | --- | --- | --- |
| 1、I am able to count on my team members for help if I have difficulties with my job. |  |  |  |  |  |  |  |
| 2、I am confident that my team members will take my interests into account when making work-related decisions. |  |  |  |  |  |  |  |
| 3、I am confident that that my team members will keep me informed about issues that concern my work. |  |  |  |  |  |  |  |
| 4、I can rely on my team members to keep their word. |  |  |  |  |  |  |  |
| 5、I trust my team members. |  |  |  |  |  |  |  |
